# Host response to cuckoo song is predicted by the future risk of brood parasitism

**DOI:** 10.1186/1742-9994-10-30

**Published:** 2013-05-22

**Authors:** Sonia Kleindorfer, Christine Evans, Diane Colombelli-Négrel, Jeremy Robertson, Matteo Griggio, Herbert Hoi

**Affiliations:** 1 School of Biological Sciences, Flinders University, Adelaide, 5042, South Australia; 2 Department of Integrative Biology and Evolution, Konrad Lorenz Institute for Ethology, University of Veterinary Medicine, Savoyenstraße 1a, Vienna, 1160, Austria

**Keywords:** Cuckoo recognition, Cuckoo threat, Risk perception, Experience, Song discrimination, Deterrent behaviour

## Abstract

**Introduction:**

Risk assessment occurs over different temporal and spatial scales and is selected for when individuals show an adaptive response to a threat. Here, we test if birds respond to the threat of brood parasitism using the acoustical cues of brood parasites in the absence of visual stimuli. We broadcast the playback of song of three brood parasites (*Chalcites* cuckoo species) and a sympatric non-parasite (striated thornbill, *Acanthiza lineata*) in the territories of superb fairy-wrens (*Malurus cyaneus*) during the peak breeding period and opportunistic breeding period. The three cuckoo species differ in brood parasite prevalence and the probability of detection by the host, which we used to rank the risk of parasitism (high risk, moderate risk, low risk).

**Results:**

Host birds showed the strongest response to the threat of cuckoo parasitism in accordance with the risk of parasitism. Resident wrens had many alarm calls and close and rapid approach to the playback speaker that was broadcasting song of the high risk brood parasite (Horsfield’s bronze-cuckoo, *C. basalis*) across the year (peak and opportunistic breeding period), some response to the moderate risk brood parasite (shining bronze-cuckoo, *C. lucidus*) during the peak breeding period, and the weakest response to the low risk brood parasite (little bronze-cuckoo, *C. minutillus*). Playback of the familiar control stimulus in wren territories evoked the least response.

**Conclusion:**

Host response to the threat of cuckoo parasitism was assessed using vocal cues of the cuckoo and was predicted by the risk of future parasitism.

## Introduction

Risk assessment and behavioural response towards a threat are important areas of individual competence that influence survival and fitness [1,2]. Throughout a lifetime, individuals are faced with a variety of threats, including infectious disease, adverse weather conditions, limited food availability, predation and brood parasitism. Both predation risk and brood parasitism have the attribute that they create a sudden threat, such as being killed or injured, or lowered reproductive success after a single moment of parasitism. Thus, individuals may be selected for different patterns of risk assessment based on the magnitude of risk and the temporal time-frame during which the threat can be detected [3,4].

Theoretically, selection should favour cues that facilitate the recognition of a costly interaction, including the degree of risk of the interaction [5]. These cues may come directly from the threat, such as the vocalisation of a predator or the sight of a brood parasite [6,7]. Or animals may evaluate indirect cues about the risk of interaction from the alarm signals of conspecifics or heterospecifics [4,7-11]. Threat recognition may be followed by the process of risk assessment, an evaluation of the situation, and an adaptive defence response. There is ample evidence that mammalian species have referential signalling for different predators [e.g. [7] and also evidence in both mammals and birds that individuals perceive and respond to the level of threat in an interaction [3,11-14]. Furthermore, the risk of a threat may change during an encounter and consequently risk assessment should also be dynamic, whereby individuals modify their response according to perceived risk as proposed by Curio and colleagues [2] and amply tested in host-parasite systems [2,15,16].

In the context of predation threat, risk assessment usually includes two risk components: risk to self of defending against the predator, and risk to offspring of being consumed by the predator. Thus, the observed defence behaviour (investment and risk taking) towards a specific predator is a result of both risk components. When the predation threat for defending parents is very high, and when parental death would result in the deaths of dependent offspring, parental risk taking should focus on parental survival. But parents could show a Kamikaze response and sacrifice their lives if offspring have a chance of survival without parental care, especially when parents have little opportunity for future reproduction [17]. For these reasons, analysis of risk assessment and response to threat in predation contexts needs to be interpreted within a life-history framework that addresses trade-offs for parents and offspring between reproductive investment and survival, and current versus future reproductive opportunity [10,18].

Brood parasites do not represent a survival risk to defending parents. Therefore, brood parasite-host systems provide ideal conditions to study risk taking and parental investment specifically in relation to brood survival while controlling for risk to parental survival [19].

There is ample evidence that parent birds show nest defence towards brood parasites. When brood parasites are in the vicinity and attempting to lay eggs, hosts have been shown to respond with mobbing or aggressive behaviour and increased vigilance at the nest [6] – and thereby lower the probability of parasitism by brood parasites [20]. Several studies have articulated how costs to the host and benefits to the brood parasite can drive the coevolution of deception and detection [13,21-23], thereby leading to complex recognition systems and discriminatory behaviour by both the host and the brood parasite [21,22,24-31]. Given that the risk of the defending parents to a secretive egg-laying brood parasite is virtually zero, the question arises whether hosts could evolve a fine tuned (dynamic) risk assessment and response towards the brood parasite adult.

Most examples of an evolutionary arms race include two combatants: one host and one parasite. But what if complex recognition systems and consequently dynamic risk assessment have more than two combatants? Here we use superb fairy-wrens (*Malurus cyaneus*) as a model species to assess risk perception in the context of multiple brood parasites. The species is suited for this investigation because superb fairy–wrens suffer multiple parasites, namely Horsfield’s bronze-cuckoo (*Chalcites basalis*) and shining bronze-cuckoo (*C. lucidus*), while little bronze-cuckoo (*C. minutillus*) is geographically allopatric and parasitises several songbird species [25]. Therefore, for the superb fairy-wren, each of these cuckoo species poses a different level of risk of brood parasitism. According to observed parasitism from previous studies, the highest risk of parasitism is from Horsfield’s bronze-cuckoo, a moderate risk of parasitism is from shining bronze-cuckoo, and the lowest risk of parasitism is from little bronze-cuckoo (see methods for details). Furthermore, superb fairy-wrens are able to acoustically discriminate (1) heterospecific alarm calls in predator contexts [32,33], (2) individually distinct alarm vocalisations of conspecifics [11,34-36], and (3) conspecific and heterospecific nestling begging calls [22,37]. Thus, we predict that wrens are able to discriminate cuckoo species that pose different levels of brood parasite risk to wrens according to the vocalisation of adult cuckoos.

Several studies have examined risk assessment in the context of host-brood parasite interactions, and have shown that hosts consistently respond to visual cues about the threat of brood parasitism [14,38]. This response includes hosts abandoning abnormal-looking eggs [21,39] and single nestlings [22,30], while the brood parasites mimic host eggs [26,40-42] and/or nestling skin coloration [38,43-45]. We have recently shown that superb fairy-wren embryos learn a begging call password while in the egg, and that parent birds use the presence or absence of the correct learned password in the nestlings’ begging call for the decision to feed or abandon the brood [37]. Clearly, it would be even more advantageous to use acoustical cues that signal brood parasite presence and increase host deterrence behaviour before egg laying. While brood parasites also produce vocalisations, few studies have tested the response to these signals by eavesdropping hosts [46,47]. Hosts that detect a brood parasite before it lays an egg could prevent it from parasitising the nest [6,48-51]. Therefore, we predict a selective advantage for potential hosts in detecting and using the song of cuckoos as they attempt to attract a mate before egg laying.

The first aim of our study is to determine whether superb fairy-wrens respond to parasite vocalisation and if the hosts are able to discriminate between varying threats represented by the different parasite species. We secondly aim to investigate whether investment into brood defence varies according to (1) the risk of brood parasitism, (2) the energetic cost of defence behaviour: we assume that alarm calls require less energy, and that approach to the threat requires more energy, and (3) the seasonal probability of parasitism risk, comparing the low risk period (opportunistic breeding by the host) and high risk period (peak breeding activity of the host). Survey data show that brood parasites have the highest song intensity during the host’s peak breeding period [52,53]. The geographically sympatric brood parasites studied here are partial migrants and some individuals remain in the territory across the year [54].

To test acoustical discrimination and investment by adult wrens towards heterospecific cuckoo songs (produced presumably by cuckoos for mate attraction and territory defence [55]), we compare host wren response as the number of alarm calls (low cost behaviour), approach distance (high cost behaviour), and latency to respond to the broadcast of a song stimulus in the wren territory during the peak (September and October) and opportunistic (April and May) breeding periods. The song stimuli used in the experimental playback trials were previously recorded from a sympatric non-parasite and three cuckoo species. Given that the threat posed by a singing brood parasite to the defending host is low, several predictions can be formulated to quantify risk assessment in the absence of threats to parental survival: (1) If wrens recognise the level of threat (cuckoo species) for offspring survival based on vocalisations, they should respond differently to the vocalisation of a threat (sympatric cuckoo species) versus the vocalisation of a non-threat (allopatric cuckoo and sympatric non-parasite, in this case striated thornbill, *Acanthiza lineata*). This prediction is about macro-level risk perception for the threat posed by brood parasites versus non-brood parasites. (2) At the micro-level of risk perception, we would expect wrens to react differently to vocalisations of different cuckoo species (species-level threats) that pose different levels of risk of brood parasitism. (3) If wrens adjust their response intensity based on the energetic costs of the defence and the risk probability, then we expect that low cost defence (alarm calls) will be observed across the year to the cuckoo species that pose the highest threat, but high cost defence (approach) would only be observed during the peak breeding period.

## Results

(a) Cuckoo response to playback of cuckoo song

Seven Horsfield’s bronze-cuckoos (high risk cuckoo) responded to the playback of Horsfield’s bronze-cuckoo song and approached within 20 m of the playback speaker during the peak breeding period (total number of trials = 71). During the opportunistic breeding period (total number of trials = 78), one Horsfield’s bronze-cuckoo responded by calling within 20 m to the conspecific playback. The shining cuckoo did not respond to our playback trials. The cuckoo response was significantly different between peak and opportunistic breeding periods (Likelihood Ratio = 5.75, *P* = 0.016).

(b) Host response to playback of cuckoo and thornbill song

**Figure 1:**
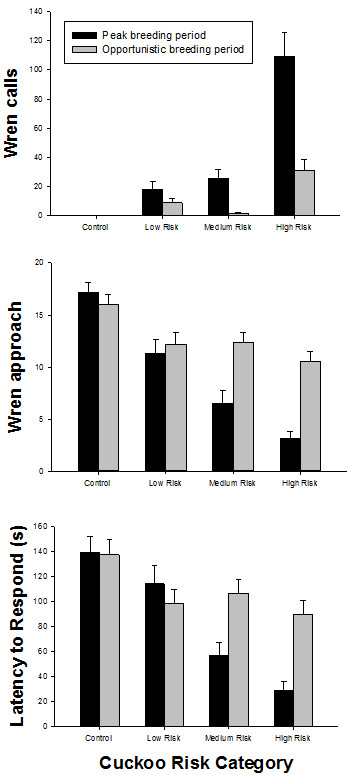
**The wren response to broadcasts of song from a control and three cuckoo species.** The results are shown as mean ± se for the following response variables: (a) the number of wren alarm calls, (b) the minimum distance (m) to approach the playback speaker, and (c) the latency (s) to respond within 10 m of the playback speaker. The song stimuli that were broadcast in the wren territories were from the following species: control = familiar non-parasite (striated thornbill), low risk = little bronze-cuckoo, moderate risk = shining bronze-cuckoo, and high risk = Horsfield’s bronze-cuckoo. We compared wren response during the peak breeding period (N = 90 playback experiments) and the opportunistic breeding period (N = 83 playback experiments). The study was done on Kangaroo Island in South Australia.

Comparing wren response to the vocalisation of brood parasites and a familiar non-parasite (striated thornbill) our results clearly revealed a stronger response towards brood parasites (MANOVA: Calls *F*_3,173_ = 14.973, *P* < 0.001; Approach *F*_3,173_ = 13.419, *P* < 0.001; Latency *F*_3,173_ = 7.582, *P* < 0.001; see Figure 1). The response to the familiar non-parasite was virtually zero and did not vary between the peak and opportunistic breeding periods (for all response behaviours *P* > 0.3). Post-hoc tests showed that wren response as calls, approach, and latency to the familiar non-parasite (control) was significantly lower in comparison to playbacks of all cuckoo species (for all *P* < 0.001).

(c) Host response to playback of cuckoo song

During the peak breeding period, wrens consistently adjusted their response to the playback of cuckoo song. Wrens had more alarm calls, a closer approach, and a shorter latency to respond to the playback of cuckoo species that had higher risk of parasitism (Figure 1, Table 1). Post-hoc pairwise comparisons (pairwise comparisons based on HSD) showed that wrens had the highest response for calls, approach, and latency to the high and intermediate risk cuckoo species (both *P* < 0.03), but no significant difference for calls to the low and intermediate risk cuckoo (both *P* > 0.1).

**Table 1:** Table 1 The response of superb fairy-wrens to the playback of cuckoo song (N=149 cuckoo playback experiments) on Kangaroo Island during the (a) opportunistic breeding period (low cuckoo risk) (N=78 trials), and (b) peak breeding period (high cuckoo risk) (N = 71 trials) of the host.

**Response variable**	**Df**	**F-value**	**P-value**
**a) Opportunistic breeding period**			
No. of Alarm Calls	2	8.67	0.001
Min. Distance to Approach (m)	2	1.01	0.34
Latency to Respond (s)	2	0.61	0.55
**b) Peak breeding period**			
No. of Alarm Calls	2	10.98	0.001
Min. Distance to Approach (m)	2	13.84	0.001
Latency to Respond (s)	2	14.44	0.001

Results are shown for a separate MANOVA test per breeding period: the predictor variable was Playback Stimulus (low risk, moderate risk, high risk cuckoo).

Opportunistic breeding period (low cuckoo risk).

During the opportunistic breeding period, wrens still responded significantly differently to the cuckoo species – but only for calls and not approach or latency (Figure 1, Table 1). Post-hoc pairwise comparisons showed significant differences in wren response for calls between high risk cuckoo and both intermediate and low risk cuckoo (both *P* < 0.01), but no difference between calls to low and intermediate risk cuckoo (*P* > 0.2). As Figure 1 shows, the number of alarm calls increased along a gradient of cuckoo risk from low to high risk cuckoo species. There were no significant pairwise differences for the other response behaviours (all *P* > 0.2).

Including cuckoo playback and breeding period within a single model and using calls, approach, and latency as fixed factors, there was a significant effect of cuckoo species and the interaction term cuckoo species × breeding period (Table 2). The significant interaction term indicates a change in the wren response pattern across breeding periods for the different cuckoo species. During the peak breeding period, wren response increased with cuckoo risk, whereas during the period of opportunistic breeding there was a weaker response pattern and only alarm calls increased with cuckoo risk category (Figure 1).

**Table 2:** A single model test of the response of superb fairy-wrens to the playback of cuckoo song (N=149 cuckoo playback experiments) on Kangaroo Island

**Factors**	**Response variable**	**Df**	**F-value**	**P-value**
Playback	No. of Alarm Calls	2	32.146	0.001
	Min. Distance to Approach (m)	2	16.743	0.001
	Latency to Respond (s)	2	10.47	0.001
Breeding Period	No. of Alarm Calls	1	37.98	0.001
	Min. Distance to Approach (m)	1	12.29	0.001
	Latency to Respond (s)	1	5.47	0.020
Playback × Breeding Period	No. of Alarm Calls	2	6.63	0.001
	Min. Distance to Approach (m)	2	5.18	0.002
	Latency to Respond (s)	2	4.94	0.002
Playback ID	No. of Alarm Calls	2	0.001	0.994
	Min. Distance to Approach (m)	2	2.43	0.121
	Latency to Respond (s)	2	3.41	0.067
Study Site	No. of Alarm Calls	2	0.30	0.583
	Min. Distance to Approach (m)	2	0.41	0.521
	Latency to Respond (s)	2	4.97	0.027

The predictor variables were playback stimulus (low risk, moderate risk, high risk cuckoo) and breeding period (peak, opportunistic). Playback ID and Study Site were entered as random effects.

## Discussion

The results of this study on host perception of a current threat in relation to the future risk of brood parasitism raise intriguing questions about adaptive risk assessment. Fairy-wrens were clearly able to discriminate sympatric and allopatric brood parasite species, as well as the control. Thus, our first prediction was supported given that the host birds showed a differentiated response across species. The second prediction was also supported given that the intensity of host response was predicted by the risk associated with the stimulus. This differentiation provides compelling evidence that fairy-wrens discriminated between particular cuckoo species (threat) – and perhaps between particular risk. The risk of cuckoo parasitism was assessed based on the probability of parasitism by the cuckoo, and the probability of detection by the host. Cuckoo species that were more likely to parasite the host and less likely to be detected were assessed as being of higher risk. Wrens responded more strongly to the playback of the high risk cuckoo than the moderate or low risk cuckoo. The strength of the response was characterised by the number of alarm calls, the approach distance, and the rapidity of response to the playback speaker (see Figure 1). The strongest response – with many alarm calls, rapid and close approach – was observed towards Horsfield´s bronze-cuckoo, which also represents the highest risk for successful brood parasitism of wren nests. The overall highest response was observed for the Horsfield´s bronze-cuckoo during the peak breeding period, when the risk of brood parasitism was also highest. Wrens had the lowest response to the allopatric cuckoo species that poses the lowest risk of parasitism but the response was still significantly higher than to the familiar control stimulus (see also [31]).

The fine-tuned response towards different cuckoo species, even species that do not occur in the area, is very surprising and raises the question of how this acoustical recognition may work. Other studies of risk perception in host-brood parasite systems have also found a strong response by hosts to the presence of brood parasites [6,48,56]. But the previous studies (1) combined visual and acoustical stimuli, and/or (2) studied the host response to one species of brood parasite [23,57-59]. In our study, host birds showed threat discrimination towards the song of several brood parasites in the absence of visual stimuli. This finding opens up many questions about the mechanism and function of heterospecific song discrimination and stimulus association in the context of risk. We discuss below how the stimulus association between song and risk perception could be shaped by learned association and experience.

There are several sources of evidence for host-brood parasite threat perception and discrimination. For example, yellow warblers (*Dendroica petechial*) showed a differentiated response to playback of intruder cowbird song based on sex: yellow warblers responded more strongly to female cowbirds (*Molothrus ater*) (they invade the host nest) than male cowbirds (they may accompany females to find host nests), and had the lowest response to the control song sparrow (*Melospiza melodia*) [48,49]. Other studies have inferred host risk perception given effects of local brood parasite density on host response sensitivity. In areas with many cuckoos, reed warbler (*Acrocephalus scirpaceus*) hosts more often mobbed the cuckoo mount placed near the nest, but tended to remain hidden in areas with few cuckoos [60]. Some of the variance in the response intensity by the host is explained by the hosts’ experience with that threat in the local population (but see [61]). In our study site, the high risk cuckoo (to which the wrens also had the highest response) was also the most common cuckoo species [62].

**Figure 2:**
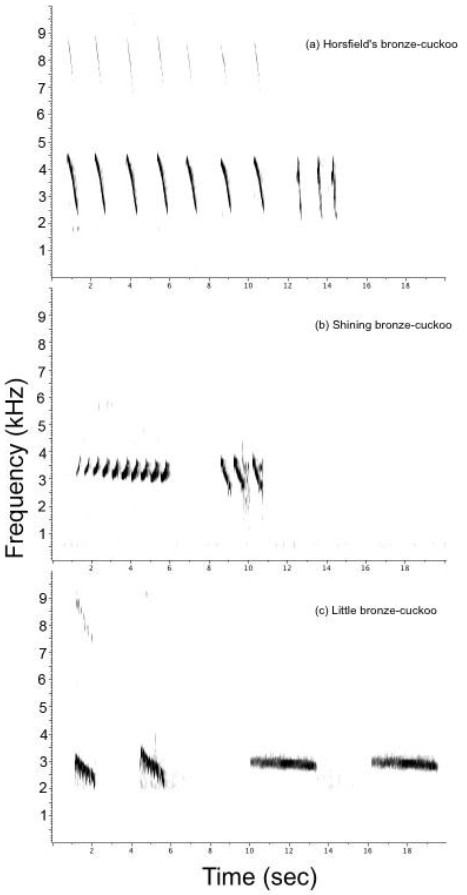
**Spectrograms of the songs of the three species of cuckoos used in the playback experiments to test host response.** The three cuckoos represent different levels of risk of brood parasitism, with the Horsfield's bronze-cuckoo representing a high risk, the shining bronze-cuckoo a moderate risk, and the little bronze-cuckoo a low risk.

An exclusive density mechanism is hard to invoke in our study, because wrens did react towards a totally unknown cuckoo species, namely the little bronze-cuckoo – and had the lowest response to the familiar non-threat, the striated thornbill (see also [63,64] . So the question arises: what other cues do birds use to distinguish the different cuckoo species? Perhaps wrens used structural characteristics in the acoustical signal as a cue for risk (Figure 2). Australian cuckoo species have different song structure, which can consist of a combination of upward and downward frequency sweeps, with occasional constant frequencies [65]. It is possible that, despite these differences between cuckoo species, some song characteristics are common to the cuckoo group. It is also possible that the wrens responded to the song structure of the cuckoo for reasons other than risk perception. Magrath and Bennett [66] recently showed that superb fairy-wrens fled in response to the playback of noisy miner alarm calls – but only when the two species shared the same location, and not in relation to call structure. In other studies, wrens responded to both structurally similar and dissimilar heterospecific aerial alarm calls if the species co-occurred in the same study area [32,33]. Combined, the results by Magrath and colleagues suggest that wrens did not respond to alarm call structure, but rather to the familiarity of the acoustical signal. Thus, similarity in acoustical signals between cuckoo species may partially explain the wren response to the allopatric cuckoo species (Figure 2), while familiarity or density cues could explain additional variation in response intensity to the sympatric cuckoo species. The similarity in cuckoo song structure is probably explained by shared phylogeny rather than cuckoo vocal mimicry. It is well known that cuckoos visually mimic the wing shape and barring plumage of predatory birds [67,68] and this mimicry has functional significance [68,69]. However it does not seem reasonable to mimic another parasite when its call elicits mobbing behaviour by a potential host.

There is a growing body of research on mechanisms of cuckoo discrimination that involve social transmission [70-72], trial-and-error learning [22,73], visual and temporal cues such as hatch order [74], and non-phenotypic discrimination cues [29]. From an ontogenic perspective, we suggest that cuckoo density could stimulate neural organisation through the frequency of singing either in embryos [37] or fledglings, and that such neural organisation could correlate with recognition of cuckoo song by adult wrens. In this scenario, we would predict that neural organisation in developing wrens would be influenced by the stimulus intensity (density) of cuckoo song in the natal breeding ground, which remains to be tested. In addition, adult hosts could have experience with cuckoos from their earlier breeding seasons, which may result in higher discrimination towards familiar stimuli [75]. This prediction can be tested by comparing the response of naive and experienced breeders [76]. But caution is required to equate age with breeding experience, given that many southern hemisphere species may postpone breeding for several years until conditions are favourable. Hence, southern hemisphere populations may have a proportion of old but inexperienced breeders.

Our third prediction about the type (low cost, high cost) of anti-parasite behaviour and the risk of brood parasitism is also supported. In this study, we compared wren response to the playback of cuckoo song during a period of high risk of cuckoo parasitism (peak breeding period) and a period of low risk of cuckoo parasitism (opportunistic breeding period). Wrens showed a differentiated response to the cuckoo species between the breeding periods (Table 2). In general, the wrens had a much lower response to all cuckoo species during the opportunistic breeding period – but they nonetheless had a higher number of alarm calls to the high risk cuckoo (Figure 1). During the peak breeding period, the wrens had a consistently higher response to all cuckoo species, with the strongest response to the high risk cuckoo, an intermediate response to the intermediate risk cuckoo, and a low response to the low risk cuckoo. Stated in terms of the purported energetic cost of defence, wrens adjusted their response according to cuckoo species for low cost behaviour (alarm calls) but not for high cost behaviour (approach). During the opportunistic breeding period, the high cost behaviour (approach) was infrequent and was not differentiated across the cuckoo species (Figure 1), but the low cost behaviour (alarm calls) was highest for the high risk cuckoo species. During the peak breeding period, the high cost behaviour (approach) was more frequent and was highest to the high risk cuckoo. The higher incidence of high cost defence behaviour during the peak breeding period is likely an adaptive response because wrens are at the greatest risk of brood parasitism at that time. Other studies have also found a temporal pattern of cuckoo defence during the breeding season – with higher nest-protection behaviour by hosts towards brood parasite mounts during the egg-laying phase or early in the breeding cycle [6,57,77]. We extended this temporal framework to test host response to the threat of a cuckoo species in their territory. The host species is long-lived, highly territorial, and sedentary year-round. We argue that defence against a brood parasite should also occur outside the peak breeding period. Sedentary and long-lived hosts are predicted to benefit if their defence behaviour results in brood parasites leaving the area or avoiding a particular area for attempted egg-laying in the future.

To test predictions about the adaptive response towards a threat, we need to observe variation in the response over a wide range of conditions. We tested host response towards a range of conditions, from (1) highest risk in the presence of a very efficient brood parasite during the main breeding period, to (2) lowest risk in the presence of a non-parasite or geographically allopatric brood parasite outside the main breeding period. We needed negative and positive controls to test if the difference in host response was explained by the experimental stimuli across a range of conditions. The non-parasite and the geographically allopatric parasite outside the breeding period were the two negative controls for comparison; the two geographically sympatric parasites across the year were the positive controls. The findings in this study support our initial predictions about dynamic risk assessment: wrens showed a plastic risk response across cuckoo species and the temporal context of the risk.

In conclusion, hosts showed a differentiated response to the threat posed by an acoustical stimulus that was associated with future risk of cuckoo parasitism. Our functional interpretation of these findings is that hosts that perceive and respond to the threat of brood parasitism before egg-laying by the cuckoo should have lower brood parasitism. Here, we show that host birds had the strongest response to the mating song of the cuckoo species that was most likely to attempt brood parasitism at their nest in the next weeks (see also [52]). This finding offers several avenues for new research focusing on the mechanism of discrimination as well as the ontogeny of discrimination. For example, what is the role of exposure, experience, and learning for the host to adaptively associate the acoustical cue – the cuckoo song – with the future risk of parasitism?

## Methods

(a) Study species

The superb fairy-wren is a small (11 g) insectivorous passerine endemic to south-eastern Australia [78,79]. The species is long-lived, sedentary, and territorial [80]. Females disperse on average 1–10 km while males remain in the natal territory or in nearby territories [80-83]. Eggs are housed in a domed nest that is usually built 0–150 cm off the ground [84]. The clutch size is two to three eggs and, depending on ecological conditions, females produce one to three clutches per year [84]. The species is characterised by a cooperative breeding system, but the number of auxiliary males per territory (0–9) differs across study populations and has been low (0–1) in South Australia since 2005 [85-88]. The species has extraordinarily high levels of extra-pair fertilisations whereby 70-95% of nests contain extra-pair young [85,89,90], but there was no significant association between the number of auxiliary males and the proportion of extra-pair young [90]. The peak breeding season in South Australia is between August-February [91]. Because the birds are sedentary and may breed if environmental conditions are favourable [80,84], we refer to the sampling period between April to May as the opportunistic breeding period. Most songbirds in the southern hemisphere are opportunistic breeders dependent on rainfall and food abundance [92]. It is for this reason that we refer to peak breeding and opportunistic breeding rather than “non-breeding”, which is a concept more appropriately applied to northern hemisphere songbirds.

(b) Cuckoo parasitism

The superb fairy-wren is a model system to study host-parasite interactions in Australia [38]. Unlike many northern hemisphere examples of brood parasite (*Cuculus canorus*) and host interactions, which are largely driven by selection for egg mimicry by cuckoos and foreign egg detection by hosts [23], the main southern hemisphere examples, including Mangrove Gerygone (*Gerygone levigaster*) and fairy-wrens, abandon foreign nestlings [14,24,25]. Here we test the acoustic discrimination by adult wrens towards three cuckoo species: Horsfield’s bronze-cuckoo, shining bronze-cuckoo, and little bronze-cuckoo. The cost of nestling discrimination rather than egg discrimination is very high, and therefore we predict that in southern hemisphere systems (especially in species with dark domed nests), hosts should be selected to identify the threat of parasitism earlier in the breeding cycle [29,75]. Horsfield’s bronze-cuckoo is the most common brood parasite in nests of superb fairy-wrens, with a range in prevalence across years from 0-37% in populations studied in Canberra [15], and 0-4% in South Australia (14 out of 232 nests) [37]; Horsfield’s bronze-cuckoo nestlings were abandoned in 38% of cases in Canberra [22] and in 83% of cases in South Australia (one out of six cuckoo nestlings was reared until fledging) ( [37], unpublished data). The Shining bronze-cuckoo was found in 5.7% of fairy-wren nests [93], but was not observed in wren nests studied in Canberra or South Australia. However, when researchers cross-fostered shining bronze-cuckoo eggs at wren nests, they were always abandoned as nestlings [22]. The two sympatric cuckoo species are partial migrants, whereby some individuals remain in the same geographical area year-round while others may have short-distance migration [54]. Little bronze-cuckoo occurs in tropical regions of northern Australia and New Guinea, but not in South Australia [62]. The little bronze-cuckoo is a common parasite of gerygone and other fairy-wren species; because it does not occur in the same geographical area as our study species, it poses virtually no risk of brood parasitism to the South Australian fairy-wrens. We hereafter refer to the cuckoo species as high risk (Horsfield’s bronze-cuckoo), moderate risk (shining bronze-cuckoo), and low risk (little bronze-cuckoo). We used the song of the striated thornbill (*Acanthiza lineata*) as the control stimulus. The striated thornbill is a small (9 g) insectivorous sympatric bird that is common across the superb fairy-wren habitat. We used the control playback of a familiar non-threat to test the idea that wren response intensity reflects risk assessment and not familiarity.

(c) Study sites

We collected data at three study sites on Kangaroo Island, South Australia: Flinders Chase National Park (35°54’S, 136°47’E), Vivonne Bay Conservation Park (36°00'S 137°09'E) and Kelly Hill Caves Conservation Park (35°59′S, 136°52′E). The study sites were separated by ~20 km, which is twice the mean dispersal distance of females. Furthermore, previous study showed little gene flow between the three sites [83].

(d) Playback stimuli

We used playback of cuckoo and thornbill song to test risk assessment by superb fairy-wrens. The cuckoo song playback trials were done in September-October 2010 (the peak breeding period) and April-May 2011 (the opportunistic breeding period) (see Table 3 for sample size per study site). The striated thornbill playback trials were done in October 2008 and 2012 (breeding season) and April 2008 and 2012 (non-breeding season).

**Table 3:** Sample size for playback trials of song (control = striated thornbill, low risk = little bronze-cuckoo, moderate risk = shining bronze-cuckoo, and high risk = Horsfield’s bronze-cuckoo) in the territories of superb fairy-wrens on Kangaroo Island during (a) the opportunistic breeding period, and (b) the peak breeding period of the host

**Location**	**Control**	**Low Risk cuckoo**	**Moderate risk**	**High risk cuckoo**
**(a) Opportunistic breeding period (low cuckoo risk)**				
Flinders Chase	4	7	7	7
Kelly Caves	4	3	3	3
Vivonne Bay	4	15	17	16
**Total**	12	25	27	26
**(b) Peak breeding period (high cuckoo risk)**				
Flinders Chase	4	9	7	8
Kelly Caves	4	9	9	9
Vivonne Bay	4	7	6	7
**Total**	12	25	22	24

The two sympatric cuckoo species have peak singing activity during July through October, which is when most mating occurs [84]. For the playback stimuli, we used the song recordings of 5 individual Horsfield’s bronze-cuckoo and 3 shining bronze-cuckoo (recorded in 2009), and 10 striated thornbills (recorded in 2008 and 2012). The sympatric cuckoo species were recorded in the study sites separated by at least 2 km. The 3 allopatric little bronze stimuli were provided by David Stewart, Nature Sounds, Queensland. We normalised the playbacks at −15 db and saved them as uncompressed 16 bit 44.1 kHz broadcast wave files (.wav) using Amadeus Pro 1.5 (Hairersoft Inc, Switzerland). We transferred the stimuli onto a FoxPro Scorpion x1-B (FoxPro Inc. U.S.A), which can be remotely triggered to start the playback trial while the researcher is hidden in vegetation 15–20 m from the playback speaker. The song stimuli consisted of 10 s of song followed by 10 s of silence, repeated three times per minute for three minutes (a total of 90 s of song and 90 s of silence per three minute trial; described below). Figure 2 shows spectrograms of the cuckoo songs used for the playback stimuli; they all have the same scale to facilitate comparison and were generated in Raven 1.4 using a Hann window function with 512 samples and 50% time overlap.

(e) Playback experiment

We colour banded a total of 122 wrens across all study sites and recorded playback response for 99 colour banded and 75 unbanded wrens. We tested each superb fairy-wren with one stimulus (either high risk, moderate risk, or low risk cuckoo, or the control striated thornbill). We used this design to ensure independence of the data. Table 3 shows the sample size for the number of playback trials per stimulus type for 78 cuckoo trials during the opportunistic breeding period and 71 cuckoo trials during the peak breeding period, and 24 thornbill trials (12 opportunistic breeding period, 12 peak breeding period). We walked three 2 km transects per study site to locate superb fairy-wren territories, which was informed by our long-term study of the area [37,94]. When we found a group within a territory, we placed the FoxPro Scorpion x1-B playback speaker 10 m from the birds. We observed the wrens for 3 minutes and recorded their vocalisations and approach to the speaker (pre-trial period). Then we commenced the trial with the playback of the song stimulus. We recorded the following behaviours during the trial: (1) the number of alarm calls (an indicator of low cost behaviour) (hereafter referred to as ‘calls’), (2) the minimum distance of approach to the speaker (m) (an indicator of high cost behaviour) (hereafter referred to as ‘approach’), and (3) the latency to respond (s) within 10 m of the speaker (an indicator of vigilance behaviour) (hereafter referred to as ‘latency’).

We also measured the number of trials at which we elicited a cuckoo response (approach to or vocalisation within 20 m of the playback speaker) from either of the two sympatric cuckoo species (Horsfield’s bronze and shining bronze-cuckoo).

(f) Statistical analysis

Results were analysed with PASW Statistics version 18 (PASW 18.0 for Windows, SPSS Inc., U.S.A). We used Chi-squared tests to analyse cuckoo response to the different playback stimuli. Except for the number of calls, the other wren response variables were normally distributed. We transformed the number of calls using ln x + 1 transformation to meet the assumptions for normality. We used MANOVA to test for wren response (calls, approach, latency) in relation to the song playback stimulus (cuckoo species) and breeding period (opportunistic, peak), the interaction term playback stimulus × breeding period, with study site and playback ID as random effects. We used post-hoc pairwise comparisons (HSD) to test the contribution of each playback stimulus for the overall variance in response intensity.

## Competing Interest

The authors declare that they have no competing interests.

## Authors’ contributions

SK and JR designed the experiments and collected data. JR prepared the playback stimuli. SK and HH analysed the data and wrote the paper. DCN and CE collected data during the opportunistic breeding period. HH and MG collected data during the peak breeding period. All authors read and approved the final version of the manuscript
